# Difference in electron microscopic findings among interstitial cystitis/bladder pain syndrome with distinct clinical and cystoscopic characteristics

**DOI:** 10.1038/s41598-021-96810-w

**Published:** 2021-08-26

**Authors:** Yu Khun Lee, Jia-Fong Jhang, Yuan-Hong Jiang, Yung-Hsiang Hsu, Han-Chen Ho, Hann-Chorng Kuo

**Affiliations:** 1 Department of Urology, Hualien Tzu Chi Hospital, Buddhist Tzu Chi Medical Foundation, and Tzu Chi University, Hualien, 970 Taiwan; 2 Department of Pathology, Hualien Tzu Chi Hospital, Buddhist Tzu Chi Medical Foundation, and Tzu Chi University, Hualien, Taiwan; 3 Department of Anatomy, Tzu Chi University, Hualien, Taiwan

**Keywords:** Bladder, Urology

## Abstract

Urothelial dysfunction may be a key pathomechanism underlying interstitial cystitis/bladder pain syndrome (IC/BPS). We therefore examined if clinical severity is associated with the extent of urothelial damage as revealed by electron microscopic (EM) analysis of biopsy tissue. One hundred IC/BPS patients were enrolled and 24 patients with stress urinary incontinence served as controls. Clinical symptoms were evaluated by visual analog scale pain score and O’Leary-Sant Symptom score. Bladder biopsies were obtained following cystoscopic hydrodistention. The presence of Hunner’s lesions and glomerulation grade after hydrodistention were recorded and patients classified as Hunner-type IC (HIC) or non-Hunner-type IC (NHIC). HIC patients exhibited more severe defects in urothelium cell layers, including greater loss of umbrella cells, umbrella cell surface uroplakin plaque, and tight junctions between adjacent umbrella cells, compared to control and NHIC groups (all *p* < 0.05). Both NHIC and HIC groups demonstrated more severe lamina propria inflammatory cell infiltration than controls (*p* = 0.011, *p* < 0.001, respectively). O’Leary-Sant Symptom scores were significantly higher among patients with more severe urothelial defects (*p* = 0.030). Thus, urothelium cell layer defects on EM are associated with greater clinical symptom severity.

## Introduction

Interstitial cystitis/bladder pain syndrome (IC/BPS) is chronic condition characterized by frequent bladder pain, pressure, and discomfort accompanied by persistent urge to void or high urination frequency in the absence of confusable diseases^1^. Although there are no standardized diagnostic criteria, estimated prevalence of IC/BPS ranges from 0.01 to 2.3%, with higher prevalence in females^2^. In the United State, high-sensitivity assessment criteria have identified IC/BPS in 6.5% of females and 1.9% of males, while higher specificity assessments have still found IC/BPS in 2.7% of females and 1.9% of males^3,4^.

The underlying pathogenesis for IC/BPS remains unclear^1^, although several studies have suggested urothelial dysfunction as a one potential mechanism^5^. For instance, increased urothelial permeability resulting from loss of surface glycosaminoglycans^6^, and significantly reduced expression levels of the tight junction proteins zonula occludens-1 (ZO-1) and adhesive junction protein have been reported in the bladder of IC/BPS patients^7^. In addition, some patients demonstrate upregulation of the purinoceptor P2X3, which can drive sensitization of bladder afferents in response to adenosine triphosphate release from the urothelium and contribute to bladder oversensitivity^8^.

According to characteristic endoscopic findings and histopathology, IC/BPS can be been divided into Hunner-type IC (HIC) and non-Hunner-type IC (NHIC) subtypes^9,10^. Chronic inflammation and epithelial denudation are the key histological features of HIC. In contrast, NHIC patients show less severe inflammatory changes and the epithelium is usually indistinguishable from that of the normal bladder^11^.

Several previous electron microscopy (EM) studies have described various ultrastructure changes in the bladder urothelium of IC/BPS patients, including defects in junctional complexes, epithelial cell pleomorphisms, loss of umbrella cells constituting the outer cell layer, loss of surface microvilli, and mast cell activation^12–14^. The loss of umbrella cells and reduced cell membrane microplicae on EM have been associated with clinical symptoms^15^. However, these studies did not distinguish changes unique to NHIC and HIC subgroups or assess the associations between such changes and cystoscopic characteristics. Therefore, the aim of the current study is to investigate differences in urothelial EM findings among IC/BPS patients with distinct clinical and cystoscopic characteristics following cystoscopic hydrodistention.

## Methods

### Patient enrolment

We prospectively enrolled IC/BPS patients admitted to our hospital for diagnostic cystoscopic hydrodistention and intravesical treatment from August 2016 to December 2019. All patients were diagnosed with IC/BPS according to the 2008 European Society for the Study of Interstitial Cystitis criteria^1^. Patients with confusable diseases such as bladder cancer, acute or chronic urinary tract infection, urolithiasis, bladder outlet obstruction, and neurogenic voiding dysfunction were excluded. Patients who had undergone urological procedures within the previous 3 months, such as cystoscopic hydrodistention or intravesical instillation of any therapeutic agent, were also excluded. Female patients with stress urinary incontinence but stable bladder function admitted for anti-incontinence surgery were offered enrolment as controls.

### Ethics declaration

The present study was approved by the institutional review board and ethics committee of Buddhist Tzu Chi General Hospital (IRB number 108-45A). All methods were performed in accordance with the relevant guidelines and regulations. Written informed consent was obtained from all patients and control subjects before enrolment.

### Diagnosis and symptom evaluation

After admission to the hospital, all patients received a comprehensive medical interview and physical examination. A 10-point visual analog scale (VAS) was used to evaluate bladder pain severity, while the O’Leary-Sant Symptom (OSS) questionnaire was used to evaluate symptoms and problems. All patients received video urodynamic studies to confirm IC/BPS diagnosis and exclude other conditions such as bladder outlet obstruction, detrusor overactivity, and neurogenic bladder dysfunction. All IC/BPS patients then underwent diagnostic cystoscopic hydrodistention under general anesthesia at an intravesical pressure of 80 cmH_2_O. Patients were subsequently classified as NHIC or HIC based on the presence of Hunner’s lesions. The maximal bladder capacity (MBC) and the grade of glomerulation (Nordling et al. classification) were also recorded^16^. Three cold-cup biopsies were obtained from the posterior bladder wall after hydrodistention, followed by adequate electrocauterized hemostasis. The specimens were prepared for EM investigation using the same protocol as described in our previous study^15^ and the defects were graded using a 4-point scale as follows: 0, normal; 1, mild defect; 2, moderate defect; 3, severe defect.

### Statistical analyses

The frequencies of cystoscopic characteristics identified by EM were compared between patient groups using the chi-square test. Patients were also stratified according to the severity of urothelial defects on EM (none, mild, or moderate vs. severe). Differences in quantitative symptom parameters (VAS, OSS, CBC, and MBC) were compared between these groups using the Independent t-test. A *p* < 0.05 (two-tailed) was considered significant for all tests. All analyses were performed using SPSS for Windows, version 16.0 (SPSS, Chicago, IL).

## Results

One hundred patients with IC/BPS (mean age, 54.9 ± 14.2 years; range, 21–86 years) and 24 control patients matched for age (66.3 ± 11.3 years, 46–87 years) were enrolled in this study. The mean VAS pain score of IC/BPS patients was 4.61 ± 2.82, mean OSS score 22.29 ± 8.39, mean MBC 728.2 ± 179.8 mL, and mean CBC was 268.7 ± 119.8 mL. Among the 100 patients, ninety-one were classified as NHIC and 9 as HIC. Compared to NHIC patients, those classified as HIC reported significantly higher VAS pain scores (7.63 ± 2.13 vs. 4.17 ± 2.64, *p* = 0.001) and OSS scores (30.67 ± 6.80 vs. 21.34 ± 8.07, *p* = 0.009) as well as lower MBC (500.0 ± 0.00 vs. 736.0 ± 177.8 mL, *p* < 0.01) (Table 1). Among the 91 NHIC patients, cystoscopic hydrodistention revealed grade 1 glomerulation hemorrhage in 38, grade 2 in 27, grade 3 in 4, and grade 4 in 4 patients.

**Table 1:** Clinical parameters of interstitial cystitis/bladder pain syndrome subtypes.

	NHIC (n = 91)	HIC (n = 9)	*p* value
VAS	4.17 ± 2.64	7.63 ± 2.13	0.001*
OSS	21.34 ± 8.07	30.67 ± 6.80	0.009*
MBC	736.02 ± 177.75	500.00 ± 0.00	< 0.001*
CBC	271.54 ± 115.92	235.86 ± 166.48	0.453

*p < 0.05 by independent samples t-test.

*NHIC* Non-Hunner’s interstitial cystitis, *HIC* Hunner’s interstitial cystitis, *VAS* visual analog score for pain, *OSS score* O’Leary-Sant Symptom score, *MBC* Maximal bladder capacity, *CBC* cystometric bladder capacity.

**Figure 1:**
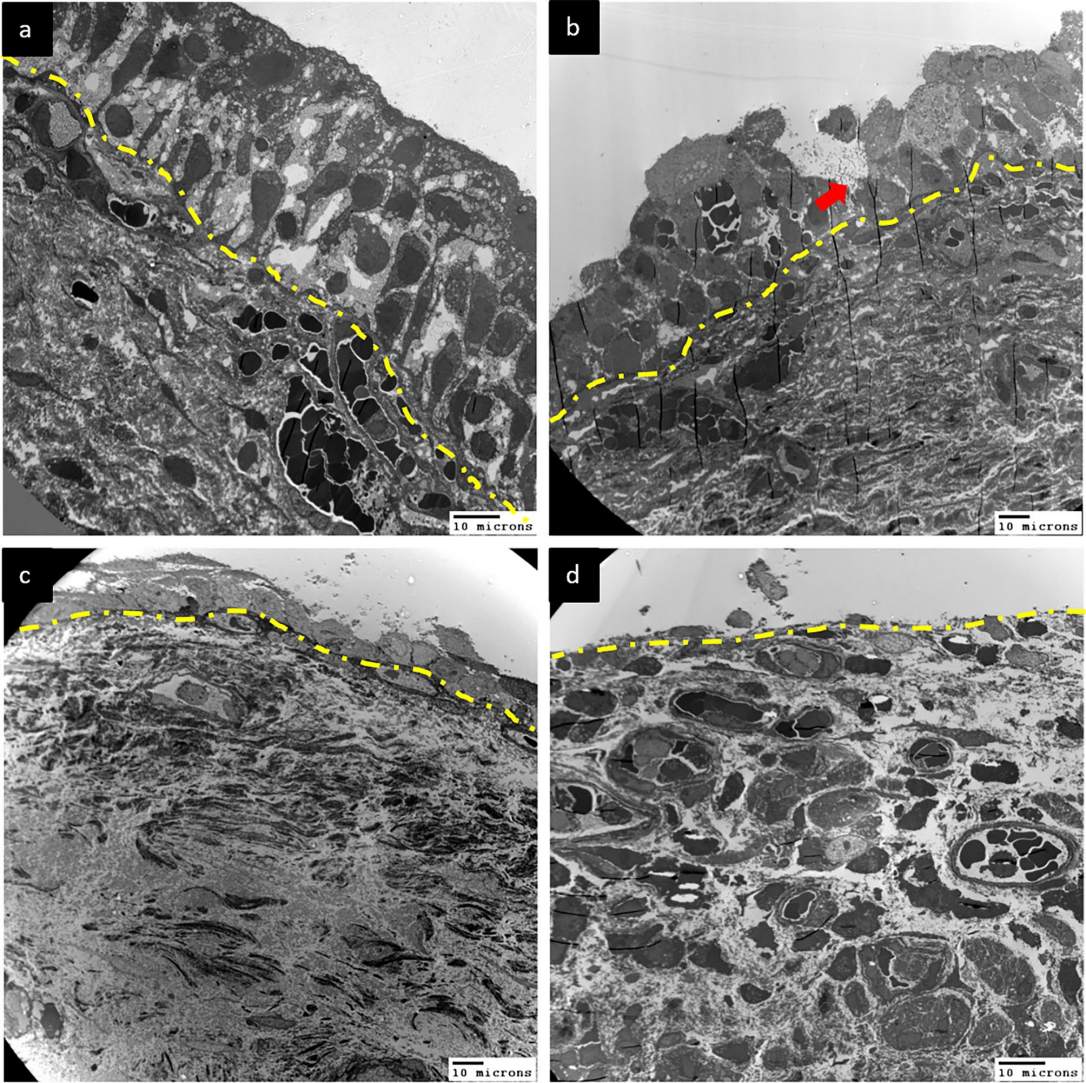
Transmission electron micrographs of the urothelium cell layer in interstitial cystitis/bladder pain syndrome (IC/BPS) patients with different grades of defect. (**a**) A sample of grade 0 (control subject) showing the normal 3–6 urothelial cell layers above the basement membrane (dotted line). (**b**) A sample of grade 1 from a IC/BPS patient showing a reduction in the number of cell layers, including modest denudation of umbrella cells (red arrows). (**c**) A grade 2 sample with only 2 or 3 urothelial cell layers remaining in most areas and more severe denudation of umbrella cells. (**d**) A grade 3 sample with almost complete loss of urothelial cells, resulting in a bare basement membrane.

**Figure 2:**
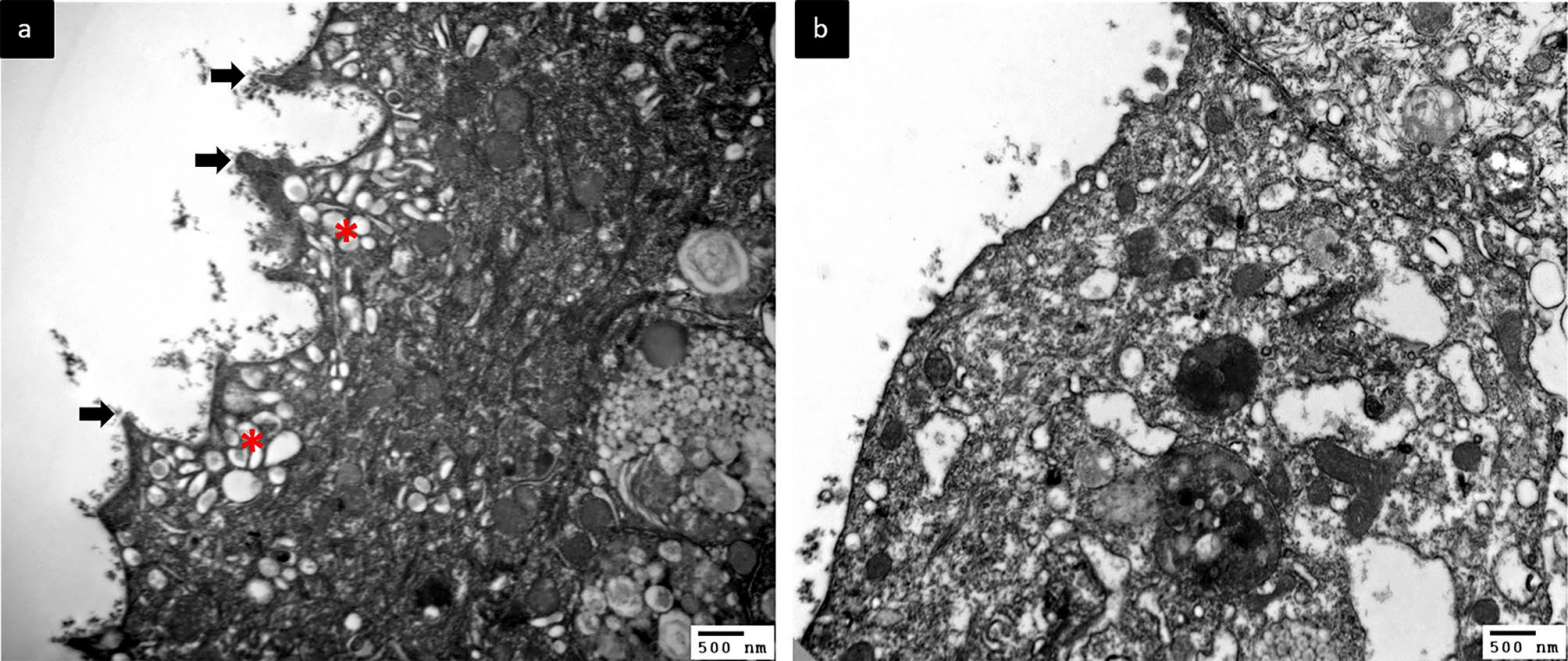
Changes in urothelial surface microstructure in IC/BPS. (**a**) A normal umbrella cell surface covered with numerous uroplakin plaques (arrows) and vesicles (asterisks). (**b**) Patient sample showing a smooth umbrella cell surface without uroplakin plaques and vesicles.

**Figure 3:**
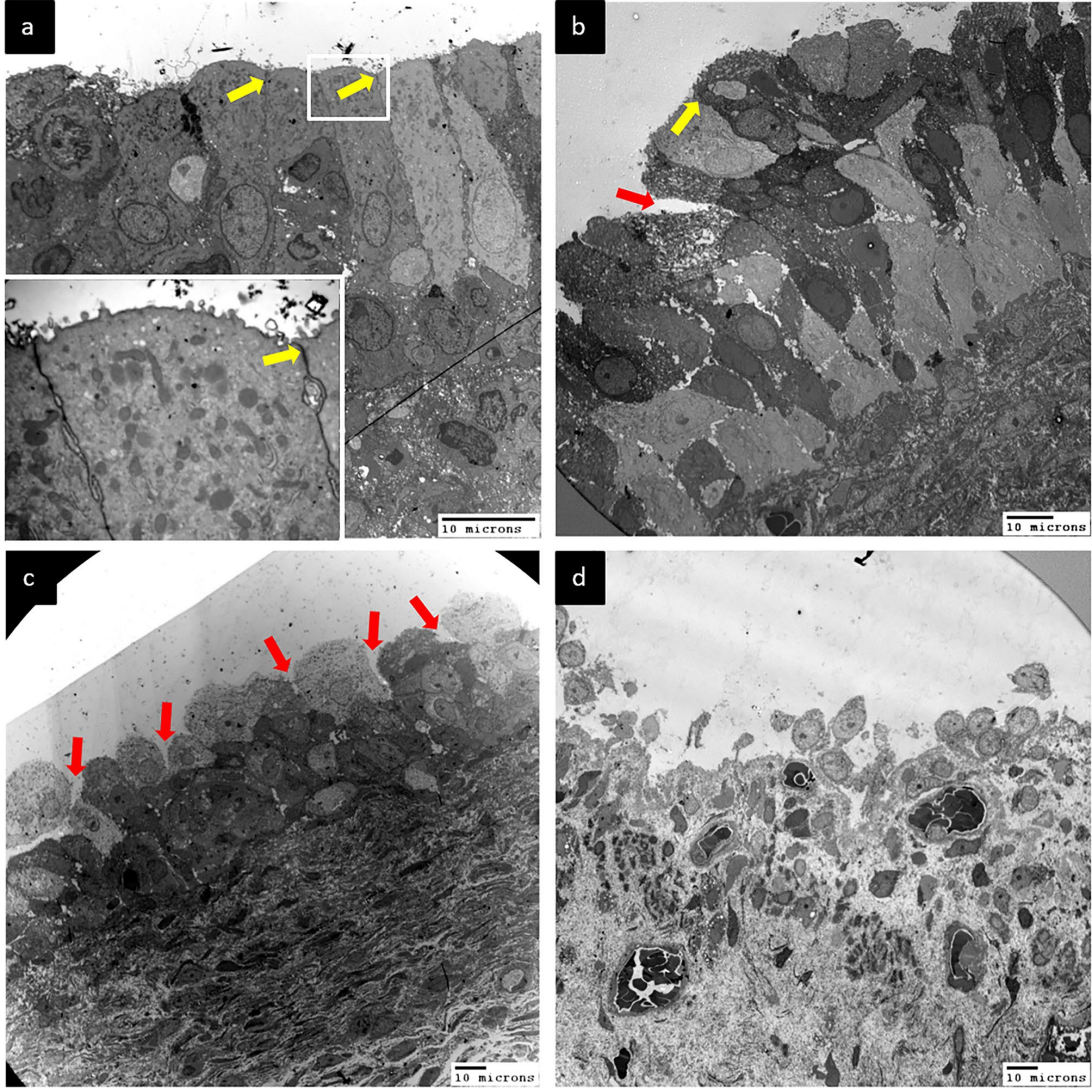
Progressive loss of urothelial tight junctions in patients with higher defect grades. (**a**) A grade 0 sample (control subject) showing a normal urothelium with ubiquitous intact tight junctions (yellow arrows). Insert shows the intact tight junction at higher magnification. (**b**) A grade 1 sample with tight junction defects (red arrows) between at least 25% of umbrella cells. (**c**) A grade 2 sample with tight junction defects between 25 and 50% of umbrella cells. (**d**) A grade 3 sample with tight junction defects between > 50% of umbrella cells.

**Figure 4:**
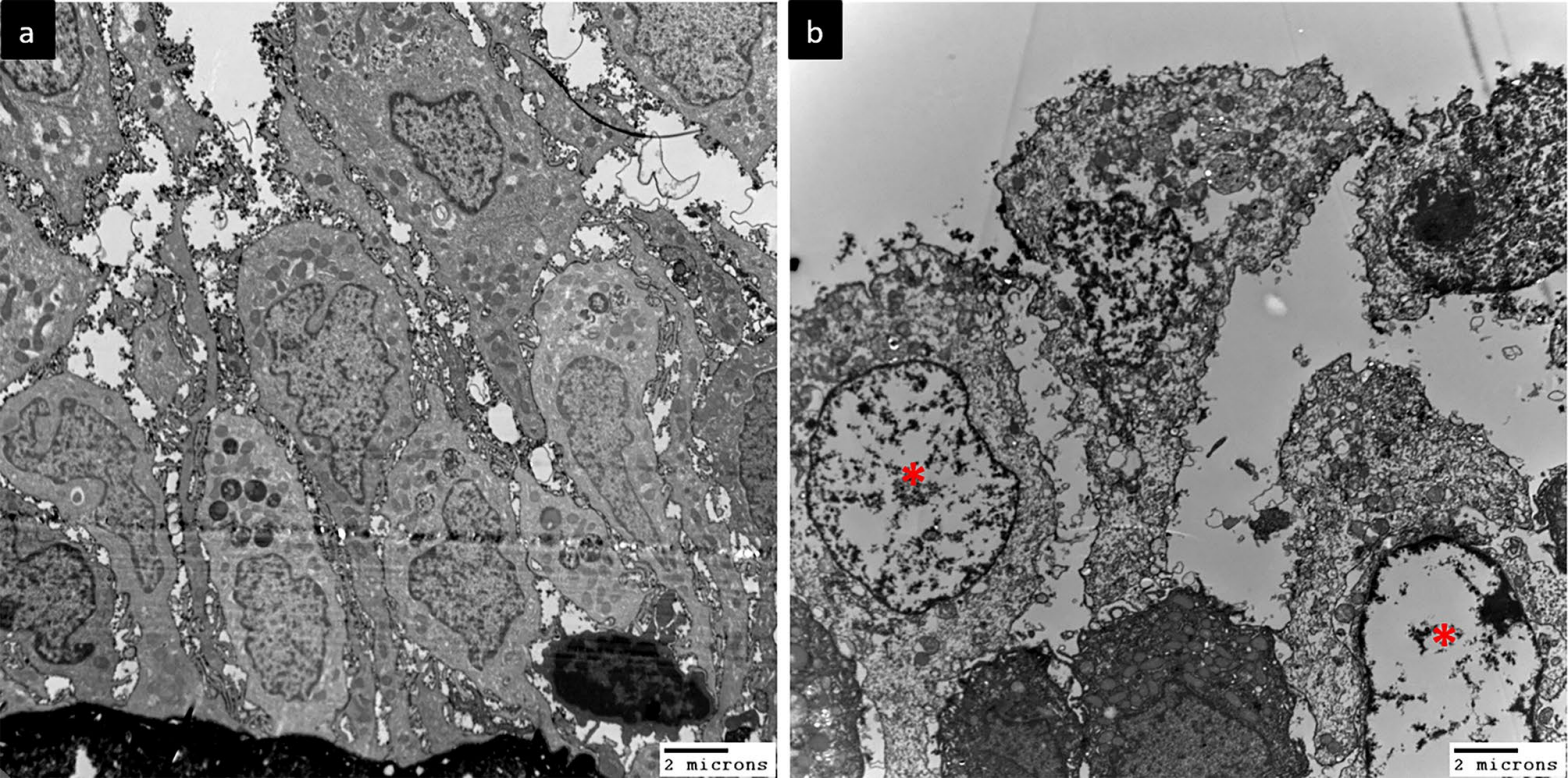
Apoptosis-like morphological changes in urothelial samples from patients. (**a**) Urothelial cells with normal organelles and nuclei. (**b**) Urothelial cells with swollen nuclei (asterisks) and ruptured organelles.

Figure 1 presents transmission EM (TEM) images of the urothelium cell layer in biopsy samples from patients with different defect grades. The normal urothelium (grade 0) consisted of 3–6 cell layers above the basement membrane with an intact umbrella (outer) cell layer (Fig. 1a). Subsequence urothelium cell layer defects grade 1 and grade 2 shown in Fig. 1b,c, while some HIC cases exhibited severe urothelium denudation and basement membrane exposure (Fig. 1d). High-power TEM of control and NHIC biopsy samples revealed that the umbrella cell surface was covered with numerous uroplakin plaques and small vesicles (Fig. 2a). In contrast, HIC patient samples exhibited a smooth umbrella cell surface without uroplakin plaques or vesicles (Fig. 2b). Epithelial layer integrity conferred by intercellular tight junctions also differed among samples from patient groups stratified by defect grades on EM (Fig. 3). Normal urothelium (grade 0) showed no gaps between adjacent umbrella cells (Fig. 3a), while patients with grades 1–3 demonstrated progressively greater tight junction defects characterized by more numerous and wider lateral interstitial spaces between adjacent umbrella cells (Fig. 3b–d). Similarly, EM findings demonstrated different abnormalities in individual urothelial cell morphology (Fig. 4), with cells of grade 0 samples exhibiting intact cytoplasmic organelles and nuclei (Fig. 4a), whereas some patient samples exhibited apoptotic urothelial cells featuring lysed organelles and swollen nuclei (Fig. 4b).

**Figure 5:**
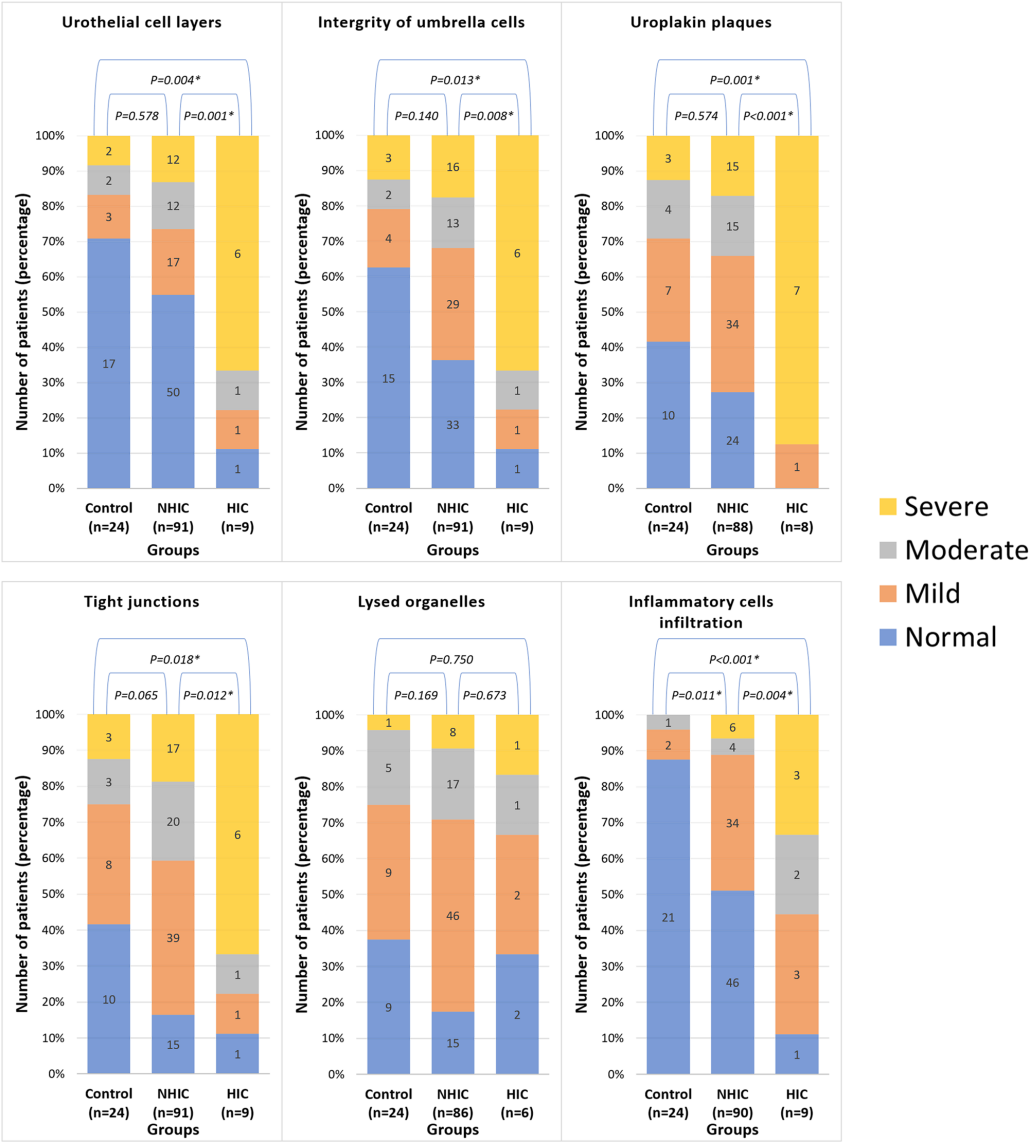
Comparison of urothelium EM defect frequencies among IC/BPS patients (HIC and NHIC) and control subjects. *p < 0.05 by chi-square test.

A comparison of these ultrastructural defects among controls and IC/BPS patient subgroups (Fig. 5) revealed more frequent urothelium cell layer defects among HIC patients compared to controls and NHIC patients (*p* = 0.004 and *p* = 0.001, respectively, by chi-square test) as well as more frequent breaches in umbrella cell layer integrity (*p* = 0.013 and *p* = 0.008, respectively), loss of umbrella cell surface uroplakin plaques (*p* = 0.001 and *p* < 0.001, respectively), and loss of tight junctions between adjacent umbrella cells (*p* = 0.018 and *p* = 0.012, respectively). Both NHIC and HIC groups exhibited more frequent lamina propria inflammatory cell infiltration compared to controls (*p* = 0.011 and *p* < 0.001, respectively), and infiltration was actually more common in the NHIC group than the HIC group (*p* = 0.004). Alternatively, the overall frequency of lysed organelles did not differ among the three groups.

Subgroup analysis of NHIC patients revealed significantly more frequent lamina propria inflammatory cell infiltration in those with severe glomerulation (*p* < 0.001) (Table 2). Further, patients with more frequent urothelium cell layer deficits also had significantly higher OSS scores (*p* = 0.030) (Table 3), while patients with more urothelial cell lysed organelles had significantly lower OSS scores (*p* = 0.003). Other associations between IC/BPS symptom parameters and EM findings are presented in Table 3.

**Table 2:** Differences in electron microscopic (EM) findings between non-Hunner’s interstitial cystitis patient.

Subgroups stratified by glomerulation grade	Mild glomerulation (grade 0–2)				Severe glomerulation (grade 3 or 4)				*p* value
EM findings	0	1	2	3	0	1	2	3	
Urothelial cell layers	47 (56.6%)	17 (20.5%)	10 (12.0%)	9 (10.8%)	3 (37.5%)	0 (0%)	2 (25.0%)	3 (37.5%)	0.072
Integrity of umbrella cells	32 (38.6%)	27 (32.5%)	11 (13.3%)	13 (15.7%)	1 (12.5%)	2 (25.0%)	2 (25.0%)	3 (37.5%)	0.243
Uroplakin plaques	23 (28.7%)	33 (41.3%)	12 (15.0%)	12 (15.0%)	1 (12.5%)	1 (12.5%)	3 (37.5%)	3 (37.5%)	0.087
Tight junctions	15 (18.1%)	37 (44.6%)	17 (20.5%)	14 (16.9%)	0 (0%)	2 (25.0%)	3 (37.5%)	3 (37.5%)	0.193
Lysed organelles	14 (17.5%)	45 (56.3%)	14 (17.5%)	7 (8.8%)	1 (16.7%)	1 (16.7%)	3 (50.0%)	1 (16.7%)	0.172
Inflammatory cells infiltration	44 (53.7%)	33 (40.2%)	1 (1.2%)	4 (4.9%)	2 (25.0%)	1 (12.5%)	3 (37.5%)	2 (25.0%)	< 0.001*

*p < 0.05 by chi-square test.

**Table 3:** Associations between IC/BPS symptom parameters and urothelial defect severity.

EM findings	Urothelial cell layers			Integrity of umbrella cells			Uroplakin plaques		
Clinical findings	Mild^†^, n = 49	Severe^†^, n = 17	*p* value	Mild^†^, n = 32	Severe^†^, n = 29	*p* value	Mild^†^, n = 23	Severe^†^, n = 33	*p* value
VAS	4.23 ± 2.51	5.63 ± 1.15	0.143	4.14 ± 2.80	5.00 ± 1.77	0.298	3.92 ± 2.75	5.11 ± 2.16	0.184
OSS	21.90 ± 6.61	27.88 ± 6.53	0.030*	21.45 ± 6.40	24.20 ± 6.99	0.235	20.75 ± 7.38	23.21 ± 6.48	0.337
MBC (ml)	707.3 ± 173.5	773.1 ± 185.7	0.201	717.2 ± 190.8	713.0 ± 145.8	0.925	719.1 ± 200.0	713.2 ± 164.8	0.147
CBC (ml)	257.9 ± 111.8	285.4 ± 68.49	0.246	287.0 ± 107.5	242.8 ± 97.1	0.106	291.2 ± 96.70	252.2 ± 92.48	0.147

EM findings	Tight junction			Lysed organelles			Inflammatory cells infiltration		
Clinical findings	Mild^†^, n = 16	Severe^†^, n = 37	*p* value	Mild^†^, n = 16	Severe^†^, n = 45	*p* value	Mild^†^, n = 45	Severe^†^, n = 34	*p* value
VAS	4.92 ± 2.60	4.62 ± 2.64	0.745	5.50 ± 2.07	4.25 ± 2.55	0.172	4.36 ± 2.66	4.44 ± 3.14	0.917
OSS	22.67 ± 6.84	23.10 ± 6.74	0.862	27.38 ± 3.54	21.29 ± 7.03	0.003*	21.78 ± 8.41	20.92 ± 8.68	0.727
MBC (ml)	686.7 ± 191.3	720.8 ± 1180.2	0.941	703.6 ± 208.0	710.2 ± 170.0	0.904	737.4 ± 173.6	722.7 ± 181.6	0.717
CBC (ml)	263.1 ± 88.9	265.4 ± 109.2	0.941	290.1 ± 84.84	267.4 ± 107.0	0.449	263.4 ± 106.8	285.5 ± 122.5	0.408

*EM* Electron microscopic, *IC/BPS* Interstitial cystitis/bladder pain syndrome, *VAS* Visual analog score for pain, *OSS score* O’Leary-Sant Symptom score, *MBC* Maximal bladder capacity, *CBC* Cystometric bladder capacity.

^†^Mild = Grade0, 1, 2; Severe = Grade 3.

*p < 0.05 by independent samples t-test.

## Discussion

In this study, we compared urothelial ultrastructural defects among NHIC, HIC, and control biopsy samples as well as between IC/BPS patient subgroups stratified by glomerulation grade. These analyses revealed more frequent or severe urothelial deficits among HIC patients than NHIC patients or control subjects as well as greater lamina propria inflammatory cell infiltration among NHIC patients with more severe glomerulation. Further, urothelial cell defects were associated with greater symptom severity according to OSS score. Collectively, these observations strongly suggest that urothelial dysfunction is a major determinant of IC/BPS clinical severity.

Interstitial cystitis/bladder pain syndrome is usually divided into NHIC and HIC subtypes according to the presence of Hunner’s lesion on cystoscopic examination^9,10^. Histological abnormalities are easily identified in HIC bladder samples, and include obvious inflammatory changes and urothelial denudation, while NHIC bladder histology is difficult to differentiate from normal bladder histology^11^. Several previous EM studies have also described pathologic changes in bladder ultrastructure among IC/BPS patients, such denudation and loss of urothelial cell layers in HIC^9,17^, as well increased apoptotic activity and urothelial barrier defects due to downregulation of tight junction proteins such as E-cadherin and ZO-1^7,18^. However, these studies did not investigate associations with clinical features or cystoscopic characteristics following hydrodistention. Here, we confirm that urothelial dysfunction progresses with disease severity. Notably, patients with HIC exhibited a greater reduction in urothelial cell layers, more extensive loss of umbrella cells, and fewer tight junctions, while the urothelial ultrastructure of NHIC patients resembled that of control subjects, underscoring the distinct pathophysiology of HIC and NHIC. The management of HIC is relatively straightforward, as many studies have reported significant pain relief after transurethral electroablation or partial cystectomy of Hunner lesions^19,20^. In contrast, the optimal management of NHIC is uncertain. Therefore, distinguishing HIC from NHIC is critical. In the current study, HIC patients always demonstrated higher clinical symptom scores and more severe urothelial defects.

Our previous EM study of IC/BPS bladder ultrastructure demonstrated that the severity of umbrella cell loss and the decrease in membrane microplicae were associated with clinical symptom severity as assessed by VAS, CBC, and MBC^15^. However, in the present larger-scale study, we found that only patients with severe urothelium cell layer deficits had significantly higher OSS scores (*p* = 0.030), possibly due to the higher ratio of NHIC to HIC patients (9:1) compared to our previous work (7:3). An ICDB (Interstitial Cystitis Database) study, demonstrated that the histopathologic features of IC/BPS bladder are highly associated with IC/BPS symptoms of nighttime frequency, urinary urgency and bladder pain^21^. These findings are consisted with current EM study.

Uroplakin plaque disruption is considered a biomarker for urothelial dysfunction in IC/BPS^5^. Disruption of uroplakin plaques destroys the integrity of the urothelial barrier and increases urothelial permeability, resulting in IC/BPS symptoms^22^. For instance, a previous study demonstrated significantly reduced Uroplakin-III in the bladders of IC/BPS patients compared to a control group^23^. Alternatively, Cho et al.^24^ found Uroplakin-III elevation in the non-ulcerative portion of HIC bladder, which may reflect a compensatory upregulation. A study of bladder samples from ulcerative lesions in HIC obtained 5 min after cystoscopic hydrodistention reported significantly lower expression levels of UP-Ia, -Ib, -II, and -III, while Uroplakin-III and III-delta4 genes were significantly upregulated in patients with NHIC^25^. In the current study, uroplakin defects were significantly more severe in HIC patients than control and NHIC groups but did not differ between control and NHIC groups. Our results support the findings of Zhen and colleagues that uroplakin expression is reduced mainly in HIC ulcer lesions, while it remains unclear if changes in uroplakin expression are also common in NHIC bladder.

Bladder permeability barrier function is maintained by the apical membrane, tight junctions between urothelial cells, and by active trafficking mechanisms^26,27^ Umbrella cells located at the apical layer of the bladder provide uroplakins for enhanced tissue flexibility, form the first tight junction-dense barrier, and contribute to active transport^5^, so processes that damage umbrella cells will lead to a dramatic loss of barrier integrity^28^. In turn, leakage of urine into the underlying suburothelial layer activates sensory receptors, resulting in irritative bladder symptoms and hypersensitivity to filling (reduced functional bladder capacity)^29^. In healthy bladder, urothelial damage is rapidly repaired, but this reparative process may be lacking or deficient in IC/BPS bladder due to subsequent neurogenic inflammation^30^. In the current study, HIC bladder exhibited the most extensive loss of umbrella cell layers, tight junctions, and uroplakin plaques, indicating greatest deficiency in barrier function and suggesting that severe chronic inflammation suppresses that capacity of umbrella cells to regenerate.

Cystoscopic hydrodistention is one of the main diagnostic tools for IC/BPS^1^.

Bladder glomerulation hemorrhage may result from excessive expression of vascular endothelial growth factor (VEGF) and concomitant generation of immature suburothelial vessels more prone to rupture during cystoscopic hydrodistention^31,32^. However, glomerulation hemorrhage after cystoscopic hydrodistention is not specific to IC/BPS, but may also be observed in other pathologies associated with chronic inflammation of the urothelium, such as urolithiasis, benign prostate hyperplasia, and stress urinary incontinence, as well as in asymptomatic patients^33^. Further, we found that glomerulation hemorrhage grade was associated with inflammatory cell infiltration in the lamina propria but not with other urothelial cell defects. Suburothelial inflammatory cell infiltration may indicate local active inflammation or injury, during which VEGF overexpression would increase vascular permeability, leading to glomerulation, edema, and further inflammation^34^.

TNF-α is a pleiotropic, pro-inflammatory cytokine released by mast cells in the bladder urothelium of patients with IC/BPS^35^. In a previous study, which using a transgenic mouse model to mimic IC/BPS, showed TNF-α were associated with the formation of urothelial lesions and loss of barrier function of the bladder^35^. Besides, TNF-α was shown to promote urothelial apoptosis and cause the characteristic symptoms of IC/BPS^36^. In the current study, we also found that urothelial defects and inflammatory cells accumulation at lamina propria and is common EM findings in IC/BPS bladder, which consistent with these prior rodent studies. The severity of inflammatory cell infiltration was associated with glomerulation hemorrhage grade after cystoscopic hydrodistention and the urothelium cell layer defects were associated with clinical symptom severity.

The main limitation of this study is lack of quantitative urothelial cell defect grading. In addition, the specimens were graded by a single investigator, which may introduce subjective bias. In addition, there was no standard biopsy site or tissue depth, and variations in these parameters may account for some of differences in EM findings, especially for HIC patients because bladder biopsy cannot be performed directly at the lesion. The effects of tissue crush during cold-cup biopsy and other preparation processes on urothelium EM features are also unclear. Future studies using more objective, quantitative methods such as immunofluorescence staining are needed to confirm the unique ultrastructural pathology of IC/BPS subtypes.

## Conclusions

The present study revealed significantly reduced urothelial cell numbers as well as loss of umbrella cell barrier integrity in bladder samples from HIC patients. Suburothelial inflammatory cell infiltration was also common among IC/BPS patients, especially those with high-grade glomerulation. These urothelium cell layer defects on EM were strongly associated with clinical symptom severity.

## Data Availability

No datasets were generated or analyzed during the current study.
